# Plant landscape characteristics of mountain traditional villages under cultural ecology: a case study of Pilin village

**DOI:** 10.1038/s41598-025-90795-6

**Published:** 2025-03-20

**Authors:** Ting Yang, Chunlan Du

**Affiliations:** School of Architecture and Urban Planning, Chongqing University, Chongqing, 400030 China

**Keywords:** Mountain traditional village, Plant landscape, Landscape characteristics, Influencing factors, Ecology, Environmental social sciences

## Abstract

With the rapid increase in urbanization, the landscape appearance of traditional village plants under the intensification of human activities is transforming. The lack of knowledge about the characteristics and values of traditional village plant landscapes has led to the “urban parkification” of rural plant landscapes, which makes the style and cultural characteristics of the village gradually fade, there is a need to sort out the characteristics of traditional village plant landscapes to conserve them. From the perspective of cultural ecology, this paper analyzed the characteristics of the plant landscape and its influencing factors in the “production-living-ecological” spaces of traditional mountain villages, selecting Pilin Village in Gaopo Miao Township, Guiyang as a case study. The results showed that Pilin Village has a stepped landscape pattern of “forest-field-village-field-river”, the spatial distribution of the plant landscape was summarized as “natural forest land-surrounding village forest-terraces-garden-farmland”, and the planting structure reflected a different layout in the vertical direction. The plant landscape in the “production-living-ecological” spaces was rich, with obvious differences in the spaces’ characteristics, but they also intersected, showing a “mutual adaptation” relationship with the environment and social humanities. The formation and development of the plant landscape characteristics and style of Pilin Village are the concentrated reflections of multiple factors in the cultural ecosystem, which is a dynamic process of continuous integration and adaptation with the environment from a macroscopic pattern to microscopic construction, reflecting the wisdom of Chinese ethnic minorities’ living environment construction. Based on the analysis of the plant landscape characteristics of Pilin Village and its influencing factors, the need for plant landscape conservation and construction in traditional mountain villages under the background of rural revitalization was determined, and the study provides some reference for contemporary village plant landscape planning, biodiversity conservation, and rural habitat construction.

## Introduction

Traditional villages are rich in historical information and living environment concepts^1^, and they record the historical memory, folk traditions, cultural ecology, and social development track of a specific geographical space in China^2^, and they are the greatest legacy of Chinese agricultural civilization^3^. However, with the acceleration of industrialization, urbanization, and rural modernization in China, traditional villages are disappearing dramatically, and the number of natural villages in China has decreased by 900,000 in only 10 years from 2000 to 2010, with an average of 80 to 100 villages disappearing every day. Therefore, the conservation of these villages and their sustainable development are vital^4,5^. China’s traditional villages are numerous, widely distributed, and distinctive, and on the whole, they are a small number in the North, more in the East, less in the West, and are concentrated in the Southwest^6,7^. The complex natural environment of mountainous areas, variable landforms, climatic characteristics, and multi-ethnic settlements result in the cultural richness and biological diversity of traditional villages^8^, which, consequently, have high research value^9^. The landscape of traditional villages is largely influenced by plants, and through the organic combination of the plant landscape and various elements, it generates a unique landscape appearance and simple and elegant cultural charm^10^, and the plant landscape has become a reflection and emotional object of the natural and cultural characteristics of the village^11^. Currently, due to a series of policies, such as rural revitalization and beautiful countryside, the living environment of traditional villages has encountered new development opportunities. However, due to the lack of knowledge of the characteristics and values of traditional village plant landscapes, urban thinking has gradually degraded the natural substrate and traditional production and living patterns of villages. Moreover, the village plant landscape design has changed toward the configuration mode of cities, leading to the degradation of the native plant vegetation of villages^10^, the disappearance of the characteristics of the regional plant landscape^12^, “urban parkification” of the plant landscape^13^, and damage to the original large hills and trees. Thus, the village plant landscape is losing its traditional texture and close connection with regional nature, which reduces the original landscape characteristics of the village with the transformation of the plant landscape and its connotation^11^. Therefore, determining the characteristics of traditional village plant landscapes is a practical need for the protection and inheritance of traditional village landscapes.

Mountain traditional village spaces are a multifunctional complex unity that is composed of ecological, production, and living spaces (“production-living-ecological” spaces)^14,15^. The deep natural and humanistic heritage of traditional mountain villages has attracted the attention of scholars since the 1980s, and currently, the protection of traditional mountain villages is mostly based on the structure of the material space, including the “village public space”^16,17^, “material cultural heritage”^18^, “settlement pattern”^19^, “cultural landscape”^20^, and other spatial entities, while the research on the evaluation of village values and sustainable utilization patterns is continuously increasing^21,22^. Plant landscape conservation is often a part of village conservation, although it is easily overlooked. For the renewal and transformation of villages, the plant landscape design imitates the surrounding cities, making the plant landscape incompatible with the simple and natural appearance of villages^23^, and the combination of multiple elements can better protect the appearance of traditional villages. Currently, the field of traditional village plant landscape research mainly focuses on the classification of township green space systems^24,25^, village vegetation conservation^26,27^, village plant species diversity^28,29^, the study of ethnic minority plant culture^30,31^, and village plant landscape configuration patterns^13^, and these studies have revealed the relationship between the village plant landscape and the environment in which it is located^11^. Although the investigation of village plant species applications based on the mountain environment has resulted in several studies^23,31^, the research on traditional village plant landscapes in mountainous areas with special geographical environments and multi-cultural characteristics is still limited.

Traditional mountain villages are a natural plant gene pool in which botanical landscapes play an important role^32^. With changes in natural, economic, and social factors, the plant landscape survives on the local land and reflects the qualities of the surrounding natural environment^11^. Moreover, plants and traditional culture are inextricably linked, and plants not only influence the emergence and formation of traditional culture but also often serve as the carrier of traditional culture and participate in its transmission and protection^33^. In terms of the interaction between culture and environment, the plant landscape in traditional villages has strong regional characteristics^34,35^. Therefore, identifying the existing plant landscape characteristics of villages can effectively help designers and policymakers to understand as much as possible about the current state of the plant landscape and its causes and, thus, to use it to guide the planning, design, scientific construction, and sustainable use of village plant landscapes^36^. Cultural ecology provides an observational perspective, which can reveal the characteristics and influencing factors of plant landscapes in traditional mountain villages, thus, providing a theoretical basis for the construction of village living environments and the protection of traditional village landscapes. Guizhou Province is the only mountainous province in China that has no plain support, and it is known as “no land is flat for three miles”. It is the most densely distributed, best preserved, and most ethnically distinctive area of traditional villages in China^9^. As previous studies have paid less attention to landscape characteristics of mountain village plants, we attempted to explore this aspect. In addition, the study of its plant landscape is of great significance for the construction of the traditional mountain village human settlements environment. Therefore, this study took place in Pilin Village, Gaopo Miao Township, Guiyang, as a case study, and, from the perspective of cultural ecology, the characteristics of the plant landscape and its influencing factors on traditional mountain villages were analyzed. The results of this study can enrich and deepen the conservation connotation of traditional villages and the value perception of village plant landscapes and provide useful insights for contemporary village plant landscape planning, biodiversity conservation, and rural living environment construction.

## Materials and methods

### Study area

**Fig. 1:**
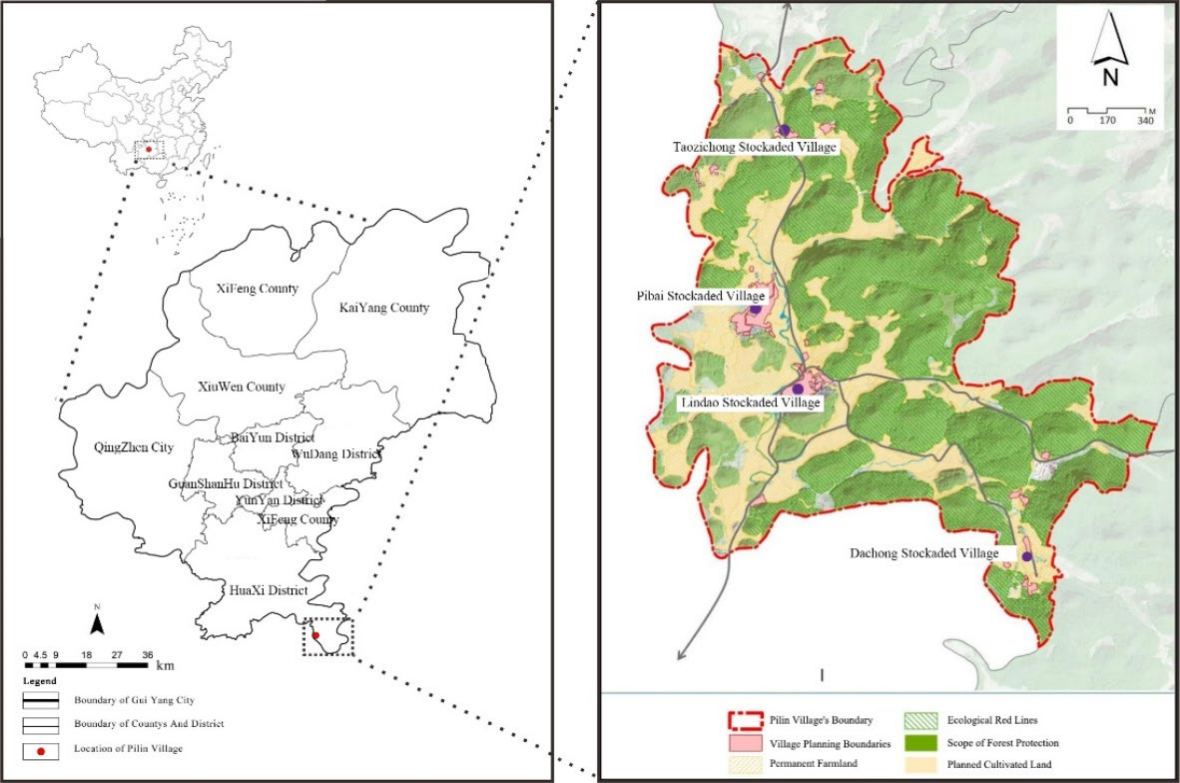
Map of the study area (image adapted from Village Plan of Pilin Village, Gaopo Township, Huaxi District, Guiyang City [2022–2035]; (https://www.huaxi.gov.cn/zwgkgb/zdlygk_5804950/cxjs/202210/t20221031_76968301.html).

Pilin Village (26º11′12′′–26º2′00′′ N, 106º47′37′′–106º53′40′′ E) is located in Gaopo Township, Huaxi District, Guiyang City (Fig. 1), and it has a karst landform type and is at an elevation of 1180–1403 m. It is mainly a mountainous landform, with the overall topography being high in the east and low in the west, extending along the north-south direction. In December 2012, it was listed as a Chinese traditional village. The village has six groups of villagers and four natural villages, with a total area of 5,270,000 m^2^. According to the “History of Yuan Dynasty”, the ancestors of Pilin Village settled here as early as the Yuan Dynasty, and after the Yuan Dynasty, a Miao-dominated settlement was gradually formed, with about 95% of the population. Miao is also known as “high mountain Miao”. The Ming dynasty records showed that the Miao “choose a cliff and cut a hole to live in, [and they lived] without a bed [and with] bamboo ladders, [which may have reached a] height [of] a hundred feet”, and the Qing Dynasty recorded that “walking along the five-foot road in Qianxi, the high mountains on the left and right sides of the road are towering, [and they are] all inhabited by Miao“^37^. The unique natural geographical environment and profound cultural and historical heritage have mutually influenced each other, forming a plant landscape with rich mountain ethnic characteristics. The village is surrounded by green hills, dense forests, a wide variety of plant species, diverse plant community types, and well-preserved plant landscapes, which are endowed with vigorous vitality and infinite charm due to natural and humanistic wisdom and have good research value.

### Data sources

1. The historical and socioeconomic data of Pilin Village are derived from local chronicles, including Huaxi District Annals of Guiyang City, A Study on Private Writings of the Miao People in Guizhou during the Qing Dynasty, and Investigation Materials on the Miao People in Guizhou. Through systematic collation, this study aims to trace the region’s folk culture, myths, and religious beliefs, and to construct a preliminary theoretical framework that explores the interaction between the village’s socioeconomic environment and its botanical landscape. Additionally, utilizing official documents such as The Village Planning of Pilin Village, Gaopo Township, Huaxi District, The 2020 Implementation Plan for the Conservation and Development of Traditional Villages and Historic Cultural Villages in Huaxi District, and the application materials for Pilin Village’s traditional village designation, the internal relationship between changes in the physical environment and the evolution of the plant landscape has been thoroughly analyzed, deepening the understanding of the cultural-ecological landscape of the village.
2. A semi-structured interview method was adopted to directly engage with villagers and collect firsthand information on the local names, folk uses, and associated legends of plants. This approach helped explore the Miao villagers’ knowledge and application of their ethnic and regional plant culture, enriching and refining the research data. Furthermore, key factors that have shaped the characteristics of the plant landscape throughout the village’s cultural development were identified. Field surveys were conducted to observe and document the spatial configuration of the current plant landscape in relation to the village’s architectural layout, water systems, and surrounding topography, as well as the practical use of various plant species. During this process, ecological plot surveys were employed to record key indicators such as the species and quantity of trees and shrubs. Complementary technologies, including drone aerial photography and hand-drawn sketches, were used to capture and present the landscape effects and configuration patterns between the village’s plant elements and environmental factors, providing comprehensive data support and visual evidence for an in-depth study of the local characteristics of the village’s plant landscape. All methods were carried out in accordance with relevant guidelines and regulations. All experimental protocols were approved by a named institutional and/or licensing committee. This study informed consent was obtained from all subjects and/or their legal guardian(s).

### Research methodology

The concept of cultural ecology arose in the 1950s^38^ and is closely related to the geographic ecology, history, and culture of a specific region^1^ as it is a dynamic accumulation of historical processes^39^ that focuses on the interrelationship between cultural formation and the environment. Regarding the characteristics and causes of the landscape, cultural ecology advocates for studying the interaction of natural, cultural, social, political, and human variables in a specific period and region, where the joint action of culture and ecology also makes the endogenous relationship of the landscape elements manifest externally as an organic combination in space^40,41^. Cultural ecology consists of three levels: the natural, economic (including production levels, production methods, and the rural economy), and social environments (including various social relations, traditional culture, and ethnic customs)^42^. Traditional village plant landscapes have local native plants that reveal the characteristics of the locality, and humans create village plant landscape spatial units that meet various requirements by understanding native plants and places and combining them with the needs of long-term production, living, settlement, and reproduction^11^, which is a spatial form of human culture, and the materialization and carrier of traditional village culture, folklore, and emotions, is a kind of cultural phenomenon^43,44^.

**Fig. 2:**
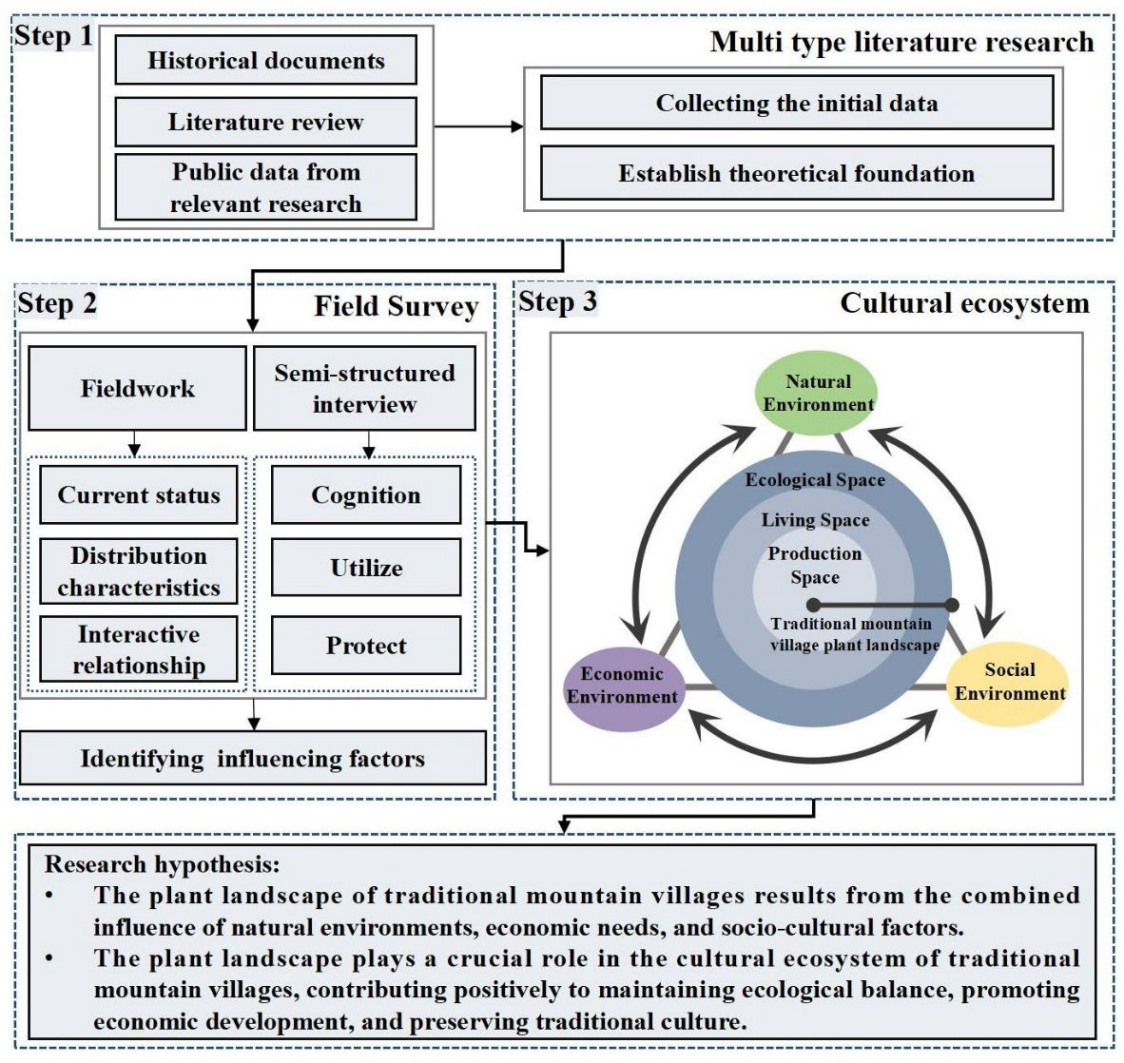
Research framework for the cultural ecosystem of the plant landscape in traditional mountain villages.

This study adopts cultural ecology as its theoretical foundation, following the three-tier analytical framework of cultural ecology to deeply explore the interaction mechanisms between the plant landscape and the natural, economic, and social environments in Pilin Village, a traditional Miao mountain village in Gaopo Township, Guiyang City. The research strategy, illustrated in Fig. 2, follows a three-step approach. The first step involves reviewing various types of literature to outline the relevant theories of cultural ecology, the current state of research on plant landscapes in traditional villages, and the historical and cultural background of Pilin Village, thus laying a solid theoretical foundation for the study. The second step employs a combination of field surveys and semi-structured interviews to conduct an in-depth investigation of the plant landscape in Pilin Village, identifying the key factors that shaped the characteristics of the plant landscape throughout the village’s cultural development. The fieldwork records the current state, distribution characteristics, and interactions of the plant landscape with its environment. Simultaneously, interviews with local villagers and village officials are used to analyze the villagers’ perception, use, and conservation attitudes toward the plant landscape. The third step centers on the interaction between the ecological, living, and productive spaces in the mountain traditional village and its plant landscape, which is identified as the core of the traditional village cultural ecosystem. Representative plant landscapes in Pilin Village, such as ancient and famous trees, surrounding village forests, and traditional residential courtyard plants, are selected for detailed case analysis. By conducting comparative analyses, the study extracts the cultural connotations, ecological values, and socioeconomic significance of Pilin Village’s plant landscape, summarizing its main characteristics and interaction mechanisms with the environment.

Based on the theoretical framework of cultural ecology, the following hypotheses are proposed: (1) The plant landscape of traditional mountain villages results from the combined influence of natural environments, economic needs, and socio-cultural factors. (2) The plant landscape plays a crucial role in the cultural ecosystem of traditional mountain villages, contributing positively to maintaining ecological balance, promoting economic development, and preserving traditional culture. Despite the study’s comprehensive and in-depth exploration of various aspects of Pilin Village’s plant landscape, several limitations remain. The limited duration of fieldwork may not fully capture all types of plant landscapes and their interactions with the environment, and the data analysis methods employed may involve some degree of subjectivity.

## Results

### Plant landscape features of Pilin village

#### Plant landscape pattern of Pilin village

##### Overall landscape pattern of the village

**Fig. 3:**
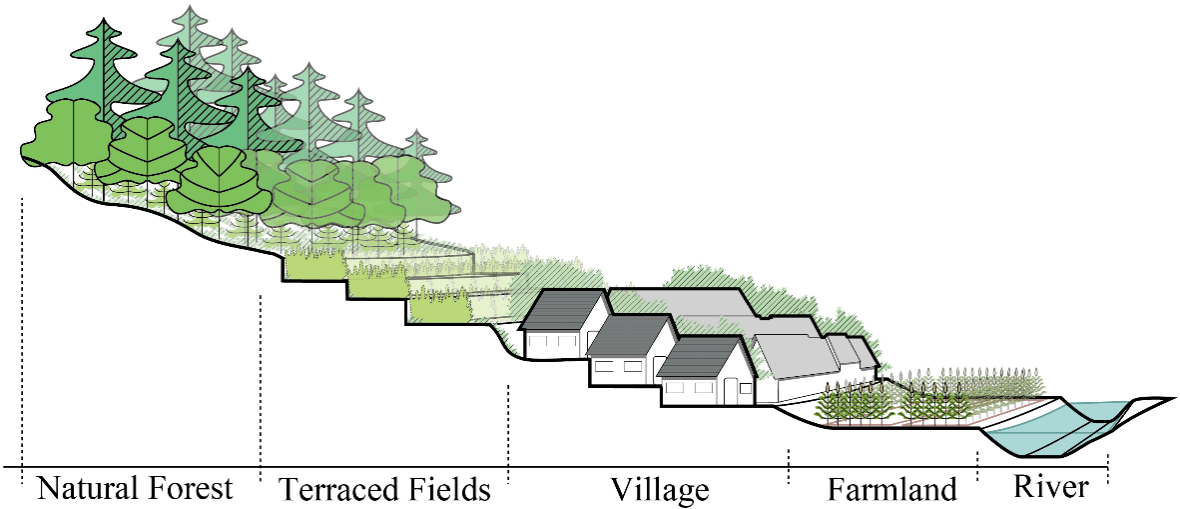
Diagram of the stepwise landscape pattern of “forest-field-village-field-river” in Pilin Village.

The site selection of Pilin Village follows the environmental concept typical of traditional mountain villages, which emphasizes the “mountains as the skeleton and water as the bloodline.” The village is situated close to nearby mountains for support, while distant mountains serve as scenic backdrops. This arrangement forms a compact and diverse landscape pattern of “forest-field-village-field-river” (Fig. 3), achieving a harmonious integration of ecology, livelihood, and production. The Miao villagers of Pilin Village live in a karst mountain environment with complex surface conditions. They have combined natural and cultural factors in selecting a terrain that faces water and backs onto mountains, building their settlement in a densely packed, stepped configuration. The orientation of village houses ensures open sightlines, creating a composite landscape pattern where production, living, and ecological spaces are interwoven. This configuration also enhances the village’s resilience to natural disasters.

##### Plant landscape plane distribution pattern

**Fig. 4:**
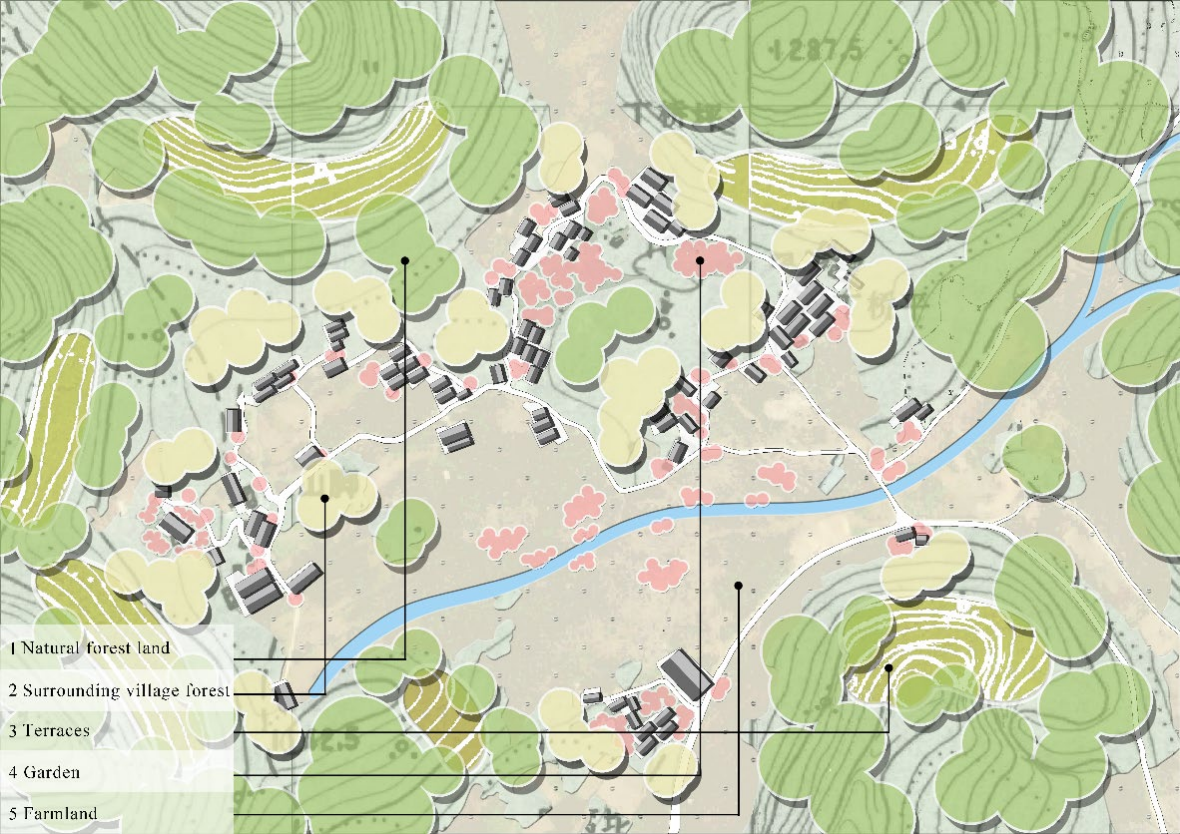
Diagram of the plant landscape plane layout of Pilin Village.

In terms of the plane layout, the plant landscape of Pilin Village mainly consisted of five types, and the spatial distribution can be summarized as “natural forest land-surrounding village forest-terraces-garden-farmland” (Fig. 4). In the village of Pilin, which is dotted with high mountains, the land for settlement is extremely limited, so the natural forest land in the ecological space has become the village’s most important part. The natural forest land in the village has become the backbone of the village landscape, providing sufficient materials for the Miao villagers. Under the influence of Miao culture and religious beliefs, the Miao villagers in Pilin Village have planted plants, such as *Neosinocalamus affinis* (Rendle) Keng f., *Cunninghamia lanceolata* (Lamb.) Hook., and *Liquidambar formosana* Hance, in the sunny places at slightly lower altitudes or around the settlement as a transition area between the village and the natural forest land, which has become the village’s protective forest. Terracing has always been a special land use method for mountain villages, has greatly improved the productivity of mountainous land in Pilin Village, and is the embodiment of the Miao paddy farming culture. The village garden was attached to the buildings as the core of the village living space, and the layout was free and random. Additionally, the farmland was located in a relatively flat position in the village, and it was mainly planted with *Zea mays* L., *Oryza sativa* Linn., and other crops, with a wide view. There was also a river running through the farmland, with rich plant species on both sides of the river bank and a beautiful landscape.

##### Vertical layout of the plant structure

Due to the different soil and hydrological conditions required for the growth of different plants, the planting structure of Pilin Village reflected a different layout in the vertical direction, showing the “mutual adaptation” relationship between the plant landscape and the environment and social and humanistic aspects (Table 1). For instance, resistant trees with a strong water-holding capacity, such as *Pinus massoniana* Lamb. and *Cunninghamia lanceolata*, grow at higher altitudes near the top of the mountains and are the main plants in the ecological space. Then, economic plants, such as *Citrus reticulata* Blanco, *Eriobotrya japonica* (Thunb.) Lindl., *Diospyros kaki* Thunb. and *Prunus salicina* Linn., were planted on the slopes at slightly lower altitudes. In addition, the gradually rising terrain of Pilin Village has laid the foundation for terraced farming, and the paddy terraces have become one of the village’s signature cultures. The Miao villagers in Pilin Village are influenced by the Chu culture to believe in witchcraft, ghosts, rituals, and nature worship, and they have planted *Liquidambar formosana*, *Cunninghamia lanceolata*, and *Neosinocalamus affinis* around the settlement as a feng shui forest or for rituals. Eleven ancient trees are listed and registered in Pilin Village, seven in Pilin Pendulum Village, and four in Lindao. The species *Alnus cremastogyne* Burk., *Celtis sinensis* Pers., and *Catalpa ovata* G. Don are sacred trees, and they are protected by the villagers as well as the ancient and famous trees. The villagers have also made full use of the scattered plots of land in front of their houses and behind the roads to plant *Brassica oleracea* Linnaeus var. *capitata* Linnaeus, *Zanthoxylum bungeanum* Maxim., *Punica granatum* Linn., *Ginkgo biloba* Linn., *Malus asiatica* Nakai, *Allium fistulosum* Linn., *Zingiber officinale* Roscoe, *Allium sativum* Linn., *Houttuynia cordata* Thunb., and other plants. In addition, *Zea mays*, *Oryza sativa*, *Brassica campestris* L., and *Solanum tuberosum* L. were grown in the farmland and are also important materials for the villagers.

**Table 1:** Vertical layout of the planting structure in Pilin village.

Land type		Main plant species
Mountaintop ↓ River Valley	Natural forest land (Ecological space)	*Pinus massoniana* Lamb.*/Cunninghamia lanceolata* (Lamb.) Hook.*/Alnus cremastogyne* Burk. */Celtis sinensis* Pers.*/ Catalpa ovata* G.Don*/Ligustrum lucidum* Ait.*/Liquidambar formosana* Hance */Engelhardia roxburghiana* Wall.*/Coriaria nepalensis* Wall.*/Prinsepia utilis* Royle etc
	Economic forest (Living space)	*Cunninghamia lanceolata* (Lamb.) Hook. */Diospyros kaki* Thunb.*/Prunus salicina* Linn. */Pyrus/Eriobotrya japonica* (Thunb.) Lindl. */Citrus reticulata* Blanco etc
	Terraces (Production space)	*Oryza sativa* Linn.
	Fengshui Forest (Living space)	*Liquidambar formosana* Hance */Cunninghamia lanceolata* (Lamb.) Hook. */Neosinocalamus affinis* (Rendle) Keng f.
	Garden (Living space)	*Osmanthus fragrans* (Thunb.) Lour.*/Brassica oleracea* Linnaeus var. *capitata* Linnaeus*/Zanthoxylum bungeanum* Maxim.*/Punica granatum* Linn. */Ginkgo biloba* Linn.*/Malus asiatica* Nakai */Allium fistulosum* Linn.*/Brassica pekinensis* (Lour.) Rupr.*/Brassica oleracea* Linnaeus var. *botrytis* Linnaeus*/Zingiber officinale* Roscoe*/Allium sativum* Linn.*/Houttuynia cordata* Thunb. etc
	Farmland (Production space)	*Zea mays* L.*/Oryza sativa* Linn. */Brassica campestris* L.*/Solanum tuberosum* L.

#### Characteristics of the plant landscape in the “production-living-ecological” spaces of Pilin village

The plant landscape of the “production-living-ecological” spaces in Pilin Village included natural jungle borders, vast terraces and farmlands, near-natural or semi-natural vegetation, thriving solitary feng shui trees, and plant landscapes that were configured by the villagers by respecting nature and their traditional culture. These elements constitute a typical and unique village plant landscape style.

##### Ecological space plant landscape

**Fig. 5:**
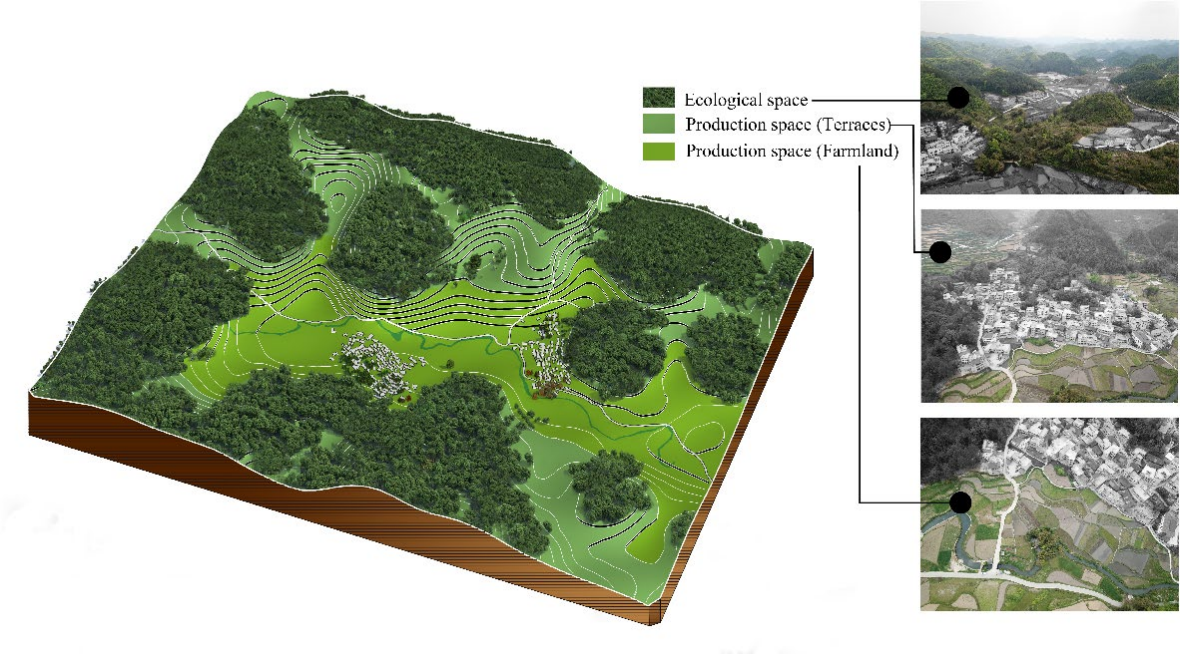
Ecological and production space plant landscape of Pilin Village.

**Fig. 6:**
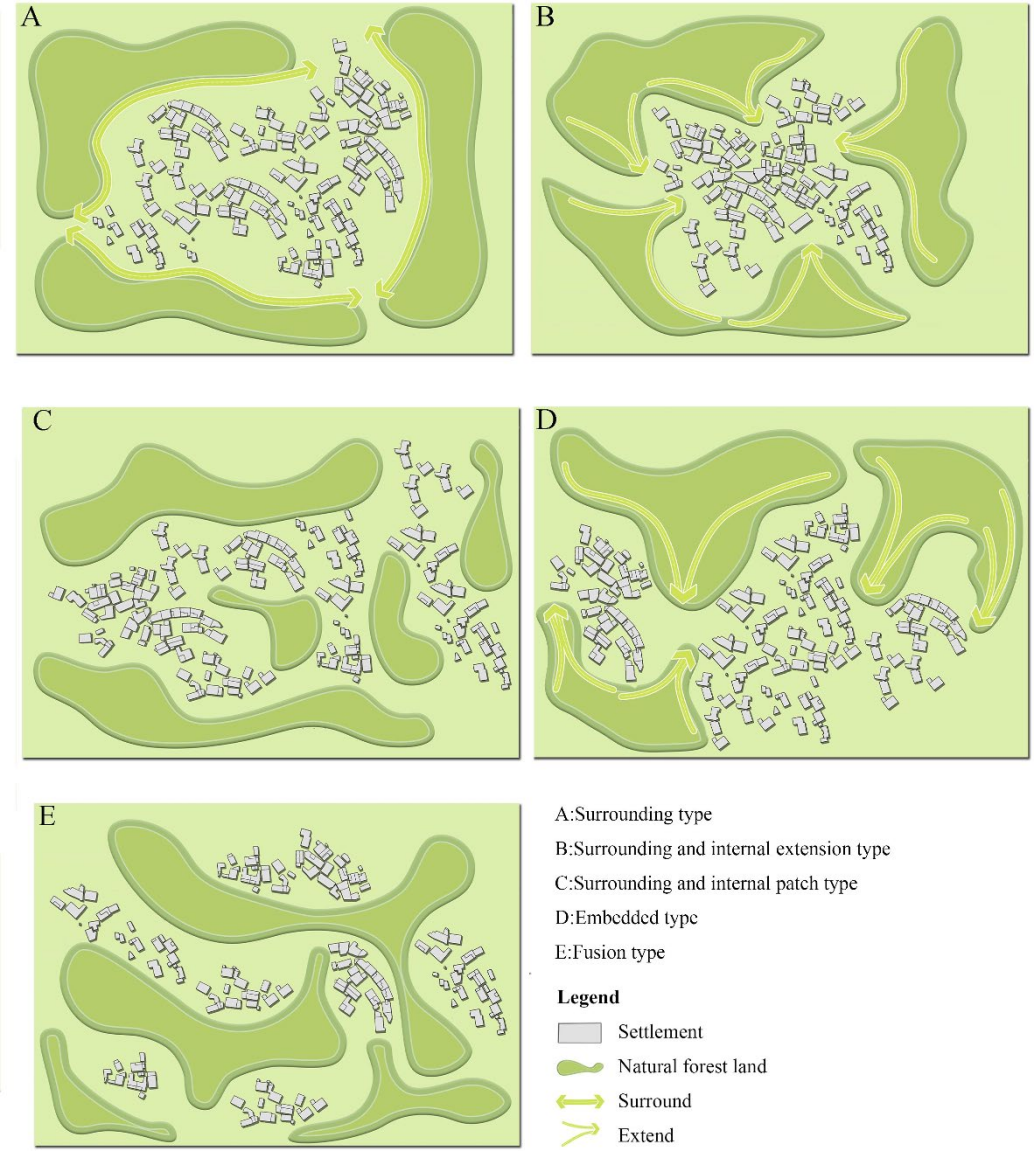
Typical spatial pattern of “natural forest land- settlements” in mountainous areas. (modified from reference^45^).

The ecological space plant landscape of traditional mountain villages is dominated by natural mountain forest, including the outer mountains, forests, and ravines that are integrated with the village layout plan, which is a “base color” and the skeleton of the overall landscape style of the village (Fig. 5). According to the differences in the natural environment in different areas, the ecological space plant landscape and the settlement form a flexible landscape space mosaic including the surrounding, embedded, and fusion types^45^ (Fig. 6). As shown in Fig. 5, the ecological space plant landscape of Pilin village was not only distributed at the periphery of the settlement but was also scattered in the middle of the settlement in the form of patches, forming a spatial pattern of a “surrounding and internal patch type”. Due to the variable topography, the ecological space of the village has rich plant species and plant landscape types, such as dense forests, shrubs, sparse forests, and grasslands, which play an important role in water conservation and the prevention of soil erosion.

##### Production space plant landscape

The production space plant landscape in mountainous traditional villages is mainly dominated by terraces and farmland (Fig. 5). The Miao people are a farming nation, and the aborigines of Pilin village built paddy terraces under the natural environment of high mountains and steep slopes, taking into account the special topography of the mountains, creating a distinctive three-dimensional agricultural landscape, and forming a farming practice that has been passed down from generation to generation, which reflects the traditional Chinese farming and reading culture of “both farming and planting, and reading my books at the same time”. Paddy terraces not only have high productivity and visual appeal but also have values, such as soil and water conservation and aesthetics. In addition, during long-term human-land interactions, the large area of agronomic crops that were planted in the fields of Pilin Village, such as *Zea mays* and *Brassica campestris*, has created a colorful open space.

##### Living space plant landscape

**Fig. 7:**
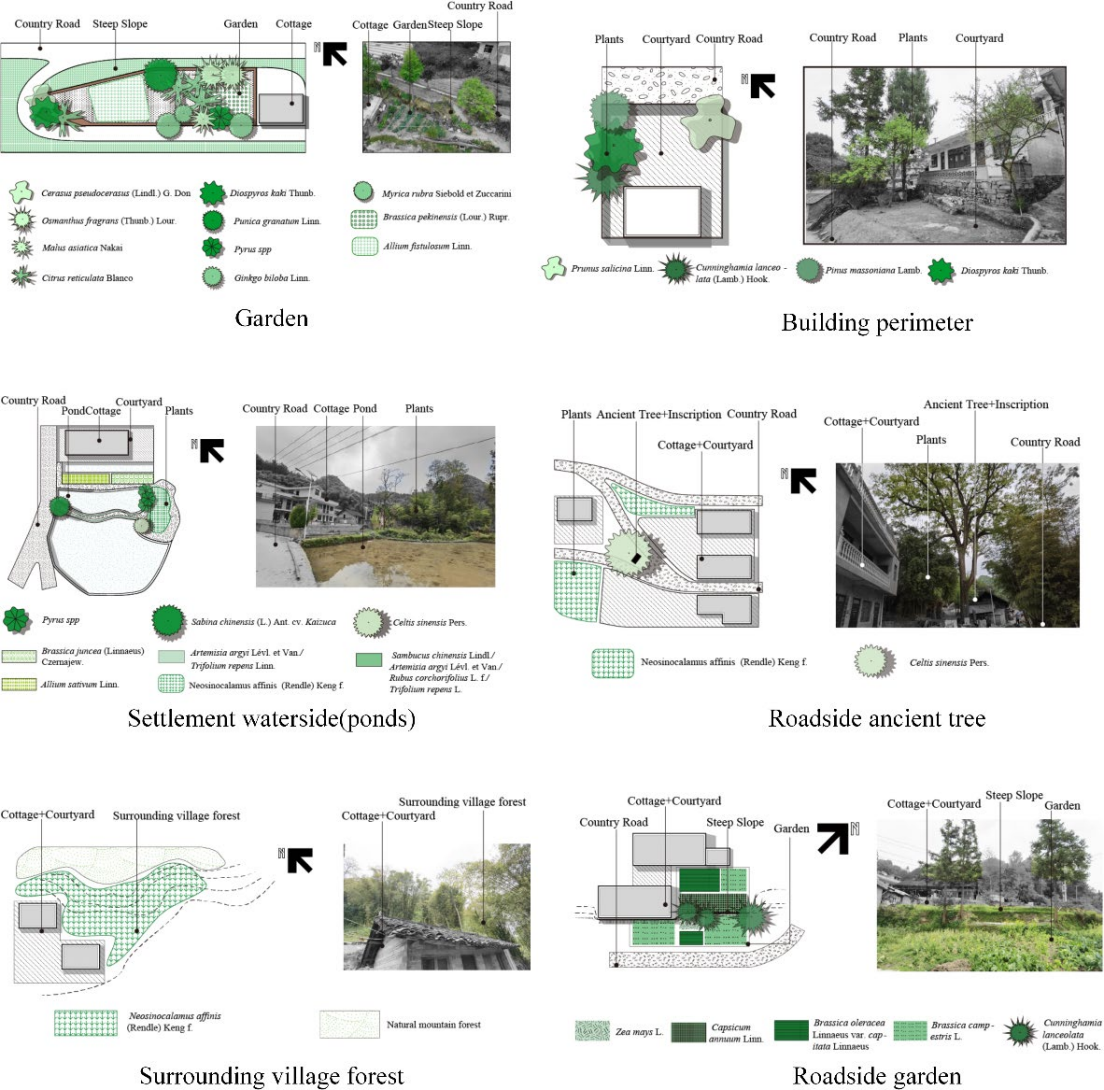
Plant landscape of the living space in Pilin Village.

The plant landscape of the living space in traditional mountain villages is dominated by gardens, and it also includes plant landscapes in four different habitats: surrounding village forest (feng shui and economic forests), settlement waterside (rivers and ponds), building perimeter (front, side, and back), and roadside (Fig. 7). The garden is an important element of the living space plant landscape in traditional mountain villages. With limited land resources in Pilin village, Miao villagers made full use of the scattered plots of different sizes and forms which located between houses and lanes to plant fruits, vegetables, and medicinal plants. In addition, the living space was rich in plant landscape types, and villagers in Pilin Village seldom occupied basic agricultural land and other ecological resources, instead making full use of the living space scattered along the hillside, foothills, forests, waterfront, and around buildings, alleyways, and other living spaces to plant fruits and vegetables, medicinal plants, economic plants, ethnic plants, and ornamental plants. The plant materials mostly came from nature. The boundary of the living space plant landscape was blurred and it intersected with every corner of the village, forming a network that linked the production, life, and residence of the village, which not only reflects the survival wisdom of the mountainous area of “less land and more people”, but also reflects the original picturesque plant landscape of the village.

### Influencing factors for the formation and development of plant landscape characteristics in Pilin village

**Fig. 8:**
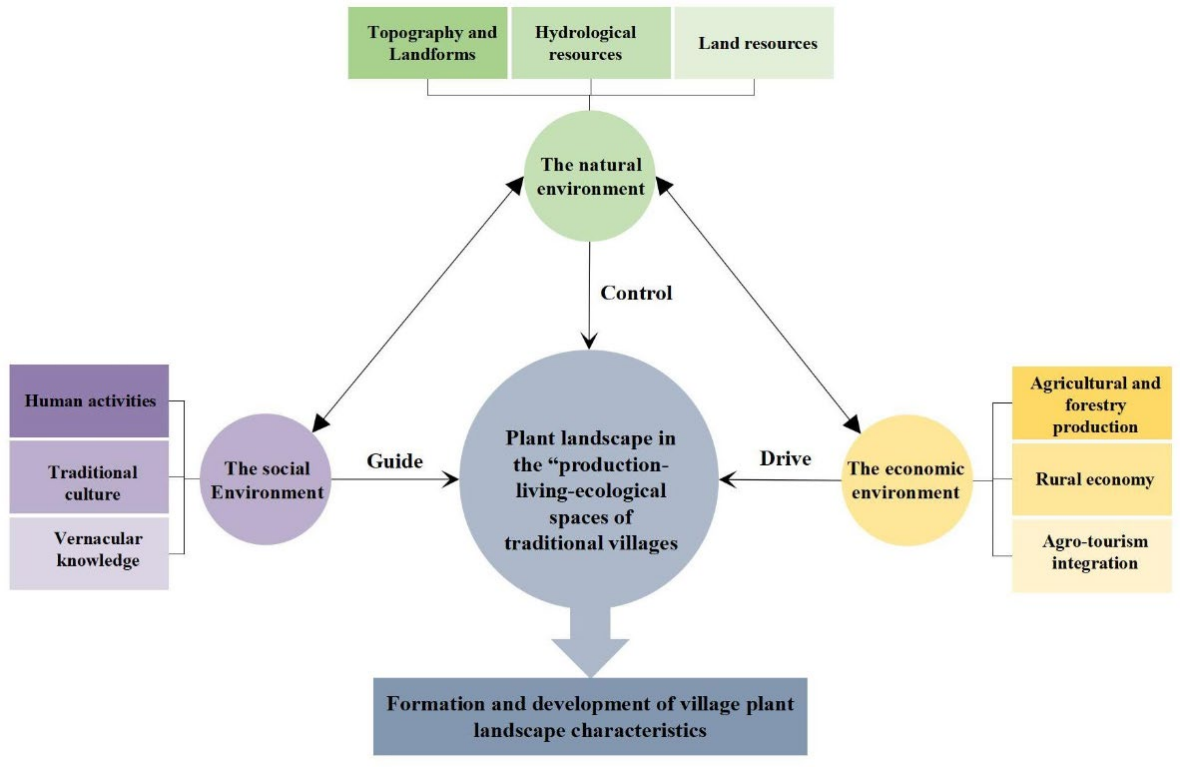
Influencing factors for the formation and development of the plant landscape characteristics of the “production-living-ecological” spaces in Pilin Village from the perspective of cultural ecology.

The formation and development of the plant landscape characteristics of the “production-living-ecological” spaces in Pilin Village is essentially a microcosm of the evolution of the habitat of traditional villages, and it is a combination of internal and external factors, which are influenced by the natural, social, and economic environment in many ways. Through the analysis of the plant landscape characteristics in the “production-living-ecological” spaces of Pilin Village, it was found that the natural environment and socio-economy of the mountainous area contributed to the plant landscape characteristics and plant application experience of Pilin Village. On the one hand, the village had rich and varied plant landscape types and characteristics in the “production-living-ecological” spaces, and the plant landscape tended to be harmonious and unified with the natural environment in the process of continuous adaptation. On the other hand, the plant landscape also developed and evolved under local social and human factors. These factors are not independent but are an integrated and complex cultural ecosystem that drives the formation and development of village plant landscape characteristics (Fig. 8).

#### The natural environment controls the basic pattern of the plant landscape in the “production-living-ecological” spaces of traditional villages

Miao villages are mostly “built on the mountain, [which is] a risky place to live” due to the strong force of nature, and the natural environment with the topography and hydrological and land resources as the core has played a fundamental role in limiting the plant landscape of the “production-living-ecological” spaces of traditional villages in the mountains. This has formed the spatial distribution form of “natural forest land-surrounding village forest-terraces-garden-farmland” in Pilin Village. Before the indigenous people of Pilin Village built the settlement, native plants occurred, forming the natural forest land of the ecological space, and the unique topography, geology, hydrology, and other natural environmental factors jointly formed its ecological habits, growth characteristics, and other essential attributes. The villagers made full use of the land, taking local materials and embracing the natural environment as a natural “fertilizer factory” to create a village plant landscape that meets the needs of daily production and life. The topography and geomorphology, as survival carriers, have a significant impact on the characteristics and appearance of the plant landscape of traditional villages in mountainous areas. The villagers of Pilin Village used contour lines to plant, cultivate, and form natural forest land, terraces, surrounding forests, and other plant landscapes, which play a vital role in protecting the fragile soil of the mountain, conserving water sources, and regulating and storing runoff. Hydrological resources are one of the most closely related elements to the plant landscape of the village. The site of Pilin Village follows the environmental pattern of “pillowing mountains, surrounding water, and facing the screen”, forming a pattern of being backed by mountains and facing the water, which can not only meet the demand for watering the farmland but can also result in a characteristic waterfront plant landscape, such as a paddy field and bamboo creek by the river. Furthermore, land resources had a significant impact on the richness of the plant landscape in Pilin Village, and the villagers have made full use of different types of land resources in the “production-living-ecological” spaces to carry out corresponding plant planting activities and production activities, forming terraces, farmlands, gardens, and various habitats (around the buildings, waterfront, and roadside). The environmental construction materials of Pilin Village and the plant configuration materials of the villagers’ living space were mostly obtained from nature, highlighting the natural and wild native beauty of the village plant landscape.

#### The social environment guides the formation of the plant landscape’s special features in the “production-living-ecological” spaces of traditional villages

The social environment plays a guiding role in the formation of the special features of the plant landscape in the “production-living-ecological” spaces of traditional villages. The social factors, such as human activities, traditional culture, and vernacular knowledge intervened in the natural background to influence the formation of the plant landscape characteristics of Pilin village. The selection of tree species in the settlement of Pilin village was mainly led by the needs of the villagers, who used contour lines for planting activities to massively transform the land, change the original ecosystem, and create a plant landscape with regional characteristics. The traditional ethnic cultures, such as “religious beliefs” (tree worship and feng shui forests) and “primitive ecological views” (forest proverbs and ancient songs in the Miao language) of Pilin Village interacted with each other to guide the formation of the characteristics of the village botanical landscape. For example, in the Miao Epic, it was written, “plant maple [with] bamboo [as] bamboo grows with the maple [and] the green grass will be at the foot of the maple”. Therefore, the maple and bamboo in Pilin Village were planted together to form a special plant landscape. The local knowledge related to agricultural and forestry production technology and management experience, such as “village regulations (forest protection regulations)”, “forest protection inscription”, and “knowledge of forest cultivation”, serves as the guaranteed mechanism of the culture and characteristics. For example, the native knowledge of forest protection and forestry cultivation, such as “there are many grasses and trees on the slope, and the paddy fields do not dry up”, “the ditch is planted with fir, the top is planted with pine, and the cypress is planted in the seam of the stone”, and “tree worship” and “bamboo worship”, means that the villagers protected the old trees in the village and planted bamboo as a courtyard forest on their own initiative. These social factors form an invisible binding force that guides the formation of the plant landscape’s special features, namely the “production-living-ecological” spaces of traditional villages, and promotes the conservation and preservation of local special plant landscapes and ancient trees. The wisdom contained therein also plays an important role in reducing natural disasters in villages, conserving water, and maintaining the sustainable development of village plant landscapes.

#### The economic environment drives the transformation of the structure and function of the plant landscape in the “production-living-ecological” spaces

In addition to the natural and social environment, the economic environment also plays an important role in the long-term development and succession of the village. Economic development is the main driving force for the structural and functional transformation of the plant landscape in the “production-living-ecological” spaces of a traditional village, and the economic factors, such as agricultural and forestry production, rural economy, and agro-tourism integration, work together in the plant landscape of the “production-living-ecological” spaces. Pilin Village has been farming for generations, and the early Miao aborigines of Pilin village made full use of the agricultural production land and continuously increased the reclamation of the mountains and steep slope areas, which has caused damage to the ecological spatial plant landscape. Additionally, the migration of young adults from the village has caused more land resources to be wasted, resulting in the abandonment of terraces and farmlands. Subsequently, policy opportunities, such as beautiful countryside construction, rural revitalization, and the selection of Pilin Village as a traditional Chinese village have injected new vitality into the development of Pilin Village. Relying on the unique mountainous paddy field resources and Miao culture to develop eco-tourism, it has promoted the revitalization and utilization of the village’s plant landscape resources. In addition, the villagers have adapted to local conditions, by using local materials and accounting for the local conditions. They have also recycled the limited mountain resources and used the plants for the construction of various buildings in the village. Furthermore, they collected fallen leaves, foliage, and bushes as green fertilizer and fodder, and they used plants such as *Cunninghamia lanceolata* and *Neosinocalamus affinis* to make reed-pipe and Miao handicraft products. Every plant and tree in front of the house is also an important source of daily medicine and food, and this forms a living space plant landscape with food, healing, and scenic functions. The villagers also plant fruit or economic forests and produce pollution-free *Zingiber officinale*, *Allium sativam* L. var. *viviparum* Regel, and vegetables near their courtyards. All these have driven the development of the village agroforestry, rural economy, and agro-tourism integration, which in turn drive the transformation of the village plant landscape structure and function.

### Suggestions for the conservation and construction of the plant landscape in traditional villages in mountainous areas under the background of rural revitalization

The plant landscape in traditional villages represents a unique cultural and ecological heritage, shaped by long-term harmonious interactions between human settlements and their natural, social, and economic environments. Distinct from urban plant landscapes, these rural configurations exhibit unique characteristics influenced by local traditions, ecological conditions, and anthropogenic activities. In the context of rural revitalization, it is crucial to adopt a holistic approach to conserve and construct these landscapes, ensuring their sustainability while respecting their cultural and ecological significance. Drawing from the analysis of Pilin Village’s plant landscape characteristics and their influencing factors, this section proposes three key suggestions: identifying the unique characteristics and cultural significance of village plant landscapes, tailoring conservation and development strategies to the specific spaces of villages, and embedding local knowledge and construction wisdom for sustainable development. These suggestions aim to provide a comprehensive framework for preserving the authenticity and vitality of traditional village plant landscapes while promoting their integration into modern rural development strategies.

The identification of unique characteristics and cultural significance of village plant landscapes constitutes a fundamental step in conservation efforts. The formation of plant landscape characteristics is the result of the interaction between people, land, nature, and culture in certain ways. It is necessary to identify the characteristics of the village plant landscape and explore the cultural stories in terms of the interactions among nature, society, the economy, and people in a specific period. This can be used to refine the life type spectrum, interspecific relationships, planting methods, and configuration patterns of the village’s regional plant landscape. During construction, ethnic plants and native trees should be used to protect the village’s style and features. Tailoring conservation and development strategies to the specific spaces of villages is another critical aspect. There are significant differences in the plant landscape characteristics and dominant functions among the “production-living-ecological” spaces. The ecology-oriented plant landscape needs minimal intervention to simulate the configuration pattern of natural or semi-natural plant communities in villages and protect the basic pattern of the plant landscape. The production-oriented plant landscape should be developed based on protection, fully explore the village plant resources, improve the planting methods, develop special agriculture. The living-oriented plant landscape should fully respect the villagers’ wishes, and the villagers should share and build together to maximize the use of the land resources to freely and randomly plant greenery wherever possible. Embedding local knowledge and construction wisdom for sustainable development is also vital. The plant landscape characteristics of Pilin Village integrate the local knowledge accumulated and comprehended by the villagers, including production techniques, plant knowledge, application management experience. This shows the construction wisdom of “adjusting measures to local conditions, obtaining materials from local sources, and using materials according to local circumstances”, which are low-cost and ecological construction methods. Therefore, traditional mountain village plant landscape construction should strive to continue the local knowledge and construction wisdom and use the village tree worship, village rules, and folk customs to enable the villagers to protect the village plant resources and special plant landscape from the bottom up. When combined with economic development, this can be used to achieve sustainable development.

## Discussion

This study, adopting a cultural ecology perspective, employs Pilin Village in Gaopo Miao Township, Guiyang, as a case study to construct a comprehensive research framework for analyzing the cultural-ecological system of plant landscapes in traditional mountain villages. Through the integration of diverse data sources, including literature reviews, semi-structured interviews, and field investigations, the study systematically identifies the distinctive characteristics and key influencing factors of plant landscapes in traditional mountain villages. Additionally, the study delineates the interactive mechanisms between Pilin Village’s plant landscape and its natural, economic, and social environments. Our findings underscore the significance of the plant landscape in traditional mountain villages as a creation of specific cultural groups^46^. The plant landscape in Pilin Village, characterized by its horizontal distribution pattern of “natural forest land-surrounding village forest-terraces-garden-farmland”, the planting structure shaped by the natural topography, and the distinctive yet interwoven characteristics across the village’s ecological, living, and productive spaces, exemplifies a “mutual adaptation” between the plant landscape and its environment. The plant landscape not only encapsulates the Miao people’s accumulated knowledge and experience in plant use but also constitutes the ecological environment most closely tied to human habitation, which is consistent with existing studies^11,33^.

Pilin Village, characterized by its complex terrain and limited land resources, exhibits a distinctive plant landscape intricately integrated into its ecological, living, and productive spaces. This plant landscape not only mirrors the cultural and ecological characteristics of the village but also encapsulates the dynamic interplay among natural, social, and economic factors within its cultural-ecological system^34,35^. The natural environment governs the basic structure of plant landscapes in the village’s three functional spaces (ecology, living, and production), the social environment shapes their distinctive features, and the economic environment drives changes in their structure and function. Conversely, the plant landscape influences the village by playing a critical role in the cultural ecological system of traditional mountain villages, impacting the generation and formation of village culture. As a cultural carrier, the plant landscape participates in the preservation and transmission of cultural heritage^47^.

In contemporary traditional villages, rapid homogenization and commercialization have led to a concerning trend: plant landscapes are increasingly resembling those of nearby urban areas, weakening the intrinsic connection between traditional village landscapes and their natural environment and regional culture, and threatening plant species diversity. Our analysis reveals that the design and construction of plant landscapes in traditional villages are often overlooked in village renewal projects and have long been neglected in the history of Chinese garden design^23,47^. To address this gap, this study aims to deepen the understanding of the characteristics and value of traditional village plant landscapes, as well as to preserve the historical culture and wisdom embedded within them. These efforts can significantly contribute to the protection of traditional knowledge, plant culture, biodiversity, and the improvement of rural living environments.

In summary, this study builds on previous research by adopting a cultural ecology perspective to investigate the characteristics and influencing factors of plant landscapes in traditional mountain villages. It enhances the comprehensiveness of identifying and uncovering rural plant landscape features and broadens the research perspective. Although exploratory in nature, the study has certain limitations. The diverse cultural aspects of traditional mountain villages, the variety of plant landscape types, and the complexity of local features pose challenges. The research approach and methodology applied to Pilin Village from a cultural ecology perspective are still somewhat limited, with exploratory processes and outcomes. Additionally, due to the challenges of conducting semi-structured interviews and fieldwork, the selection of typical plant landscape cases may be subjective, and it was not possible to identify all plant landscape features in the village. The issues of in situ conservation and innovative development of rural plant landscape characteristics still need to be explored more deeply in subsequent studies, and we hope to stimulate more discussions and collaborations aimed at preserving the unique cultural and ecological heritage of traditional villages around the world.

## Conclusion

This study analyzed the plant landscape characteristics and their influencing factors in the village’s “production-living-ecological” spaces of Pilin Village, Gaopo Miao Township, Guiyang, from a cultural ecology perspective. The village exhibited a stepped landscape pattern characterized by “forest-field-village-field-river,” with plant spatial distribution summarized as “natural forest land-surrounding village forest-terraces-garden-farmland.” The vertical planting structure displayed distinct layouts, and the “production-living-ecological” spaces were rich in plant landscape types, showing both differences and intersections in their characteristics. The natural, economic, and social environments collectively form the cultural ecosystem of the plant landscape in traditional mountain villages. The natural environment dictates the basic pattern of the plant landscape, while the social environment influences the formation of its unique features. The economic environment drives the transformation of the landscape’s structure and function. Based on the analysis of the plant landscape characteristics of Pilin Village and its influencing factors, this study provides recommendations for the conservation and construction of the plant landscape in traditional villages in mountainous areas, particularly in the context of rural revitalization.

## Data Availability

Data Availability Statement: Data is contained within the article.
